# LitR directly upregulates autoinducer synthesis and luminescence in *Aliivibrio logei*

**DOI:** 10.7717/peerj.12030

**Published:** 2021-09-21

**Authors:** Sergey Bazhenov, Olga Melkina, Vadim Fomin, Ekaterina Scheglova, Pavel Krasnik, Svetlana Khrulnova, Gennadii Zavilgelsky, Ilya Manukhov

**Affiliations:** 1 Laboratory for Molecular Genetics, Moscow Institute of Physics and Technology, Dolgoprudny, Russia; 2 Higher School of Economics, Moscow, Russia; 3 State Research Institute of Genetics and Selection of Industrial Microorganisms of the National Research Center “Kurchatov Institute”, Moscow, Russia; 4 State Research Institute of Genetics and Selection of Industrial Microorganisms of the National Research Centre “Kurchatov Institute”, Kurchatov Genomic Center, Moscow, Russia; 5 National Research Center for Hematology, Moscow, Russia

**Keywords:** Quorum sensing, *litR*, *Aliivibrio*, *luxR*, Transcriptional regulation, Luminescence

## Abstract

LitR is a master-regulator of transcription in the *ainS/R* and *luxS/PQ* quorum sensing (QS) systems of bacteria from *Vibrio* and *Aliivibrio* genera. Here, we for the first time directly investigated the influence of LitR on gene expression in the *luxI/R* QS system of psychrophilic bacteria *Aliivibrio logei*. Investigated promoters were fused with *Photorhabdus luminescens luxCDABE* reporter genes cassette in a heterological system of *Escherichia coli* cells, *litR A. logei* was introduced into the cells under control of P*_lac_* promoter. LitR has been shown to upregulate genes of autoinducer synthase (*luxI*), luciferase and reductase (*luxCDABE*), and this effect doesn’t depend on presence of *luxR* gene. To a much lesser degree, LitR induces *luxR1*, but not the *luxR2 — *the main *luxI/R* regulator. Enhanced *litR* expression leads to an increase in a LuxI-autoinducer synthesis and a subsequent LuxR-mediated activation of the *luxI/R* QS system. Effect of LitR on *luxI* transcription depends on *lux*-box sequence in *luxI* promoter even in absence of *luxR* (*lux*-box is binding site of LuxR). The last finding indicates a direct interaction of LitR with the promoter in the *lux*-box region. Investigation of the effect of LitR *A. logei* on *luxI/R* QS systems of mesophilic *Aliivibrio fischeri* and psychrophilic *Aliivibrio salmonicida* showed direct *luxR*-independent upregulation of *luxI* and *luxCDABE* genes. To a lesser degree, it induces *luxR A. fischeri* and *luxR1 A. salmonicida*. Therefore, we assume that the main role of LitR in cross-interaction of these three QS systems is stimulating the expression of *luxI*.

## Introduction

**Figure 1:**
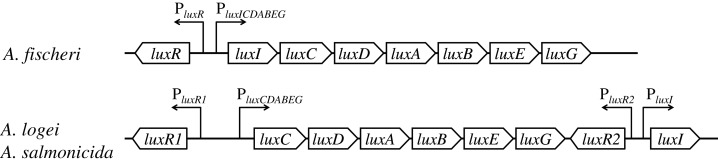
Structural organization of *lux* regulons of bacteria from *Aliivibrio* genus (Manukhov et al., 2011). The *luxA* and *luxB* genes encode α- and β-subunits of the luciferase, the *luxC*, *luxD*, and *luxE* genes encode subunits of the reductase, the *luxR*, *luxR1* and *luxR2* genes encode regulatory proteins, activators of *lux* operon transcription, and *luxI* encodes LuxI synthase, which produces autoinducer (AI). Promoters with their directions of transcription are indicated by angular arrows with the letter P.

In *lux* regulons of some *Aliivibrio* bacteria, such as *Aliivibrio fischeri*, the regulatory gene *luxR* and structural genes *luxICDABEG* are divergently transcribed from a pair of promoters situated between them; the main regulatory operator is *lux-*box (the site of LuxR binding) (Devine, Countryman & Baldwin, 1988; Devine et al., 1989; Meighen, 1991; Stevens, Dolan & Greenberg, 1994). Structures of *lux* regulons of mesophilic and psychrophilic bacteria of the *Aliivibrio* genus significantly differ: *lux* regulons of psychrophilic ones, *Aliivibrio logei* and *Aliivibrio salmonicida*, are divided into two groups of genes: *luxR1-luxCDABEG* and *luxR2-luxI* (Fig. 1) (Fidopiastis, Sørum & Ruby, 1999; Khrulnova et al., 2010; Manukhov et al., 2011).

*luxR* and *luxI* genes constitute a quorum sensing (QS) system. *luxI-luxR* system provides regulation of the expression of *lux* regulon genes (cell luminescence control) in dependence on cell culture density (Nealson & Hastings, 1979; Fuqua, Winans & Greenberg, 1994; Zavilgelsky & Manukhov, 2001). There are several proteins, which can modulate this QS system’s work. H-NS and CRP regulate the transcription of *luxR* genes (Dunlap & Greenberg, 1988; Ulitzur et al., 1997; Melkina, Goryanin & Zavilgelsky, 2017; Melkina et al., 2019). The content of the native protein LuxR is post translationally regulated by GroEL/ES chaperons and the Lon protease (Dolan & Greenberg, 1992b; Zavil’gel’skiĭ & Manukhov, 1994; Manukhov, Kotova & Zavil’gel’skiĭ, 2006; Manukhov et al., 2010), in psychrophilic bacteria it is so for the LuxR2 protein, but not the LuxR1(Khrulnova et al., 2016; Konopleva et al., 2016).

LitR in *A. fischeri*, being the master regulator of the *ainS/R* and *luxS/PQ* QS systems, can positively regulate the *luxI/R* system by activation of a *luxR* transcription (Fidopiastis et al., 2002; Miyashiro & Ruby, 2012). The deletion of the *litR* gene in *A. salmonicida* leads to an autoinducer (AI, 3-oxo-C6-homoserine lactone) concentration decrease detected at late growth phase (Hansen et al., 2015). It is known that *Vibrio harveyi* LuxR, which is a close homologue to LitR from *A. fischeri*, *A. logei* and *A. salmonicida*, does not require binding to signaling molecules (Fidopiastis et al., 2002; Rutherford et al., 2011). In dependence on its own concentration in the cell *V. harveyi* LuxR binds to the DNA in promoter regions and activates transcription by a direct interaction with RNA-polymerase (Rutherford et al., 2011; Van Kessel et al., 2013). The concentration of LitR is an object of LuxO-mediated regulation by the *luxS/PQ* and *ainS/R* QS systems and it increases when these QS pathways are activated (Miyashiro et al., 2010; Rutherford et al., 2011; Van Kessel et al., 2013).

Thus, the effect of LitR enhanced expression on the *lux* promoters after activation of *ainS/R* and *luxS/PQ* QS systems was investigated in mesophilic bacteria *A. fischeri*, but not the psychrophilic ones (*A. logei*, *A. salmonicida*), which have other *lux* regulon structure. Previously it was supposed that *A. salmonicida* LitR could activate the *luxR1, luxR2* and *luxI* genes promoters (Khider et al., 2019), but it was not tested experimentally. The aim of this study was to determine whether there is a direct effect of *litR* on the expression of *lux* regulon of psychrophilic bacteria *A. logei* and *A. salmonicida*, and, if there is such an effect, to determine the mechanism of the effect of *litR* on proteins or promoters of *lux* regulon. The effect of *A. logei* LitR on the expression of regulatory *luxR1* and *luxR2* and structural *luxCDABEG* and *luxI* genes from *A. logei* and *A. salmonicida* was investigated in a heterologous model on *Escherichia coli* cells. Promoters of the *A. fischeri lux* genes were used as a control.

## Materials and Methods

### Bacterial strains and plasmids

All experiments were conducted with the *E. coli* MG1655 F^-^, *rph*^−1^ strain (Guyer et al., 1981), which were obtained from VKPM collection (Russia). Plasmids used in this study are listed in Table 1 and illustrated in Fig. S1. Primers and a detailed description of plasmids constructed in this study are presented in a Supplemental File (see Plasmid constructions). Genes and promoters of *Aliivibrio* bacteria were from *A. fischeri* MGU-6, *A. logei* KCh1 (Khrulnova et al., 2010) and *A. salmonicida* NCIMB 2262^T^ (provided by Jesus L. Romalde (Egidius, Wiik & Andersen, 1986)).

**Table 1:** List of plasmids, which were used in this study.

Plasmid	Relevant characteristics	Source or References
p15Tc-lac	Vector for cloning of regulatory gene under control of P_*lac*_; p15 origin, *lacI*, Tc^r^	Bazhenov et al. (2021)
p15Tc-litR	*litR* gene of *A. logei* under control of P_*lac*_; p15 origin, *lacI*, Tc^r^	This study
pDEW201	Promoter probe vector with promoterless operon *Photorhabdus luminescens luxCDABE* genes and the replication origin of pBR322; Ap^r^	Van Dyk & Rosson (1998)
pDEW201-derivative biosensor plasmids with *luxCDABE*-reporter for promoters investigation:		
pALR1	*lux*-reporter for *A. logei* P_*luxR1*_ + *luxR1*	Melkina et al. (2019)
pDewP2rev	*lux*-reporter for *A. logei* P_*luxR2*_	This study
pIVA	*lux*-reporter for *A. logei* P_*luxCDABEG*_ + *luxR1*	Khrulnova et al. (2016)
pSV16	*lux*-reporter for *A. logei* P_*luxI*_ + *luxR2*	Khrul’nova, Manukhov & Zavil’gel’skiĭ (2011)
pAS1	*lux*-reporter for *A. salmonicida* P_*luxCDABEG*_ + *luxR1*	This study
pAS2	*lux*-reporter for *A. salmonicida* P_*luxI*_ + *luxR2*	This study
pR2	*lux*-reporter for *A. logei* P_*luxI*_ without *luxR2*	Bazhenov et al. (2021)
pAFR	*lux*-reporter for *A. fischeri* P_*luxR*_ + *luxR*	Melkina et al. (2019)
pVFR1	*lux*-reporter for *A. fischeri* P_*luxICDABEG*_ + *luxR*	Manukhov, Kotova & Zavil’gel’skiĭ (2006)
pD-lb1	*lux*-reporter for *A. logei* P_*luxCDABEG*_	This study
pD-lb2	*lux*-reporter for *A. logei* P_*luxCDABEG*_ with changed *lux*-box (matches *lux*-box of *luxI* promoter)	This study

**Note:**

Ap^r^, ampicillin resistant; Tc^r^, tetracycline resistant.

### Culture media and growth conditions

Bacteria were grown in Luria-Bertani (LB) broth. The liquid LB medium was composed of 1% tryptone, 0.5% yeast extract and 0.5% NaCl, for the preparation of solid medium, agar was added to a final concentration of 1.5% m/v. The medium was supplemented with appropriate antibiotics: 100 μg/ml ampicillin, 10 μg/ml tetracycline, or their composition. Overnight cultures were grown at 37 °C with continuous agitation and then were used to inoculate liquid LB. The resulting cultures were grown at 22–25 °C with continuous agitation (200 rpm). The optical density (OD) of cell suspensions was measured with a KFK-3 photometer (Zagorsk Optical-Mechanical Plant, Russia). An induction of the *litR* gene expression (plasmid p15Tc-litR) was made at the early exponential growth phase (OD about 0.1) by addition of IPTG (isopropyl β-D-1-thiogalactopyranoside) to a final concentration of one mM.

### DNA isolation, restriction, ligation and transformation

Plasmid DNA was isolated by alkaline lysis. Cell transformation with hybrid plasmids followed standard protocols. Endonuclease restriction, DNA fragment ligation, agarose gel electrophoresis and isolation of DNA fragments from agarose gel were performed according to Green & Sambrook (2012). Restriction and ligation reactions were carried out using enzymes from Promega (USA): EcoRI, BamHI and KpnI restriction enzymes and T4 DNA ligase were used for constructing new plasmids (see Supplemental File for details).

### Chemical substances

Isopropyl β-D-1-thiogalactopyranoside (IPTG) was from Anatrace (USA).

### Measurement of bioluminescence

Bioluminescence intensities were measured in volume of 200 μl in plastic microtubes placed in front of a photomultiplier photocathode at room temperature using Biotox-7MB (BioPhisTech, Russia) or in 96-well plates using SynergyHT (Biotek Instruments, Winooski, VT, USA). Luminescence values were expressed in relative light units (RLU).

### Determination of LitR-dependent promoter regulation

The effect of LitR on the expression of genes from *lux* regulons of the *Aliivibrio* genus bacteria was investigated for three species: *A. fischeri, A. logei* and *A. salmonicida*. For this purpose we used the heterological system of *E. coli* cells transformed with different biosensor plasmids from a set (Table 1, Fig. S1) that carry *luxCDABE* genes of *P. luminescens* transcriptionally fused with promoters of interest. To introduce the *litR* gene from *A. logei* into this system, the p15Tc-litR plasmid was constructed. It comprises a p15 origin, the *litR A. logei* gene under a *lac* promoter, and a *lacI* gene, which lowers the base transcription from P_*lac*_ and allows regulating *litR* with IPTG.

### Luminescence induction factor calculation

The culture of *E. coli* cells carrying the *A. logei litR* gene under the IPTG-inducible promoter on the p15Tc-litR plasmid and the *P. luminescens luxCDABE* genes under control of the promoter of interest on a biosensor plasmid was grown to OD~0.1 in liquid LB medium, then divided into two equal parts: “control” and “induced”. The “induced” portion was supplemented with one mM IPTG. Further, the cultures were grown under equal conditions with periodic measurements of optical density and luminescence. The induction factor is the induced/control ratio of cell cultures’ luminescence. All figures show representative kinetic curves of biological independent triplicates.

### Statistics

Error bars at the graphs are the SD of three independent experiment replications.

### Computer analysis

Amino acid sequences alignment was performed using the Vector NTI software (Thermo Fisher Scientific, Waltham, MA, USA). The intrinsic disorder of proteins was predicted by their aa sequences with “Predictor of Naturally Disordered Regions” (pondr.com), one can find description of VL-XT, VSL2, VL3 and Charge-Hydropathy algorithms in Obradovic et al. (2003).

## Results

### Effect of LitR *A. logei* on promoters of regulatory genes *luxR*, *luxR1* and *luxR2*

**Figure 2:**
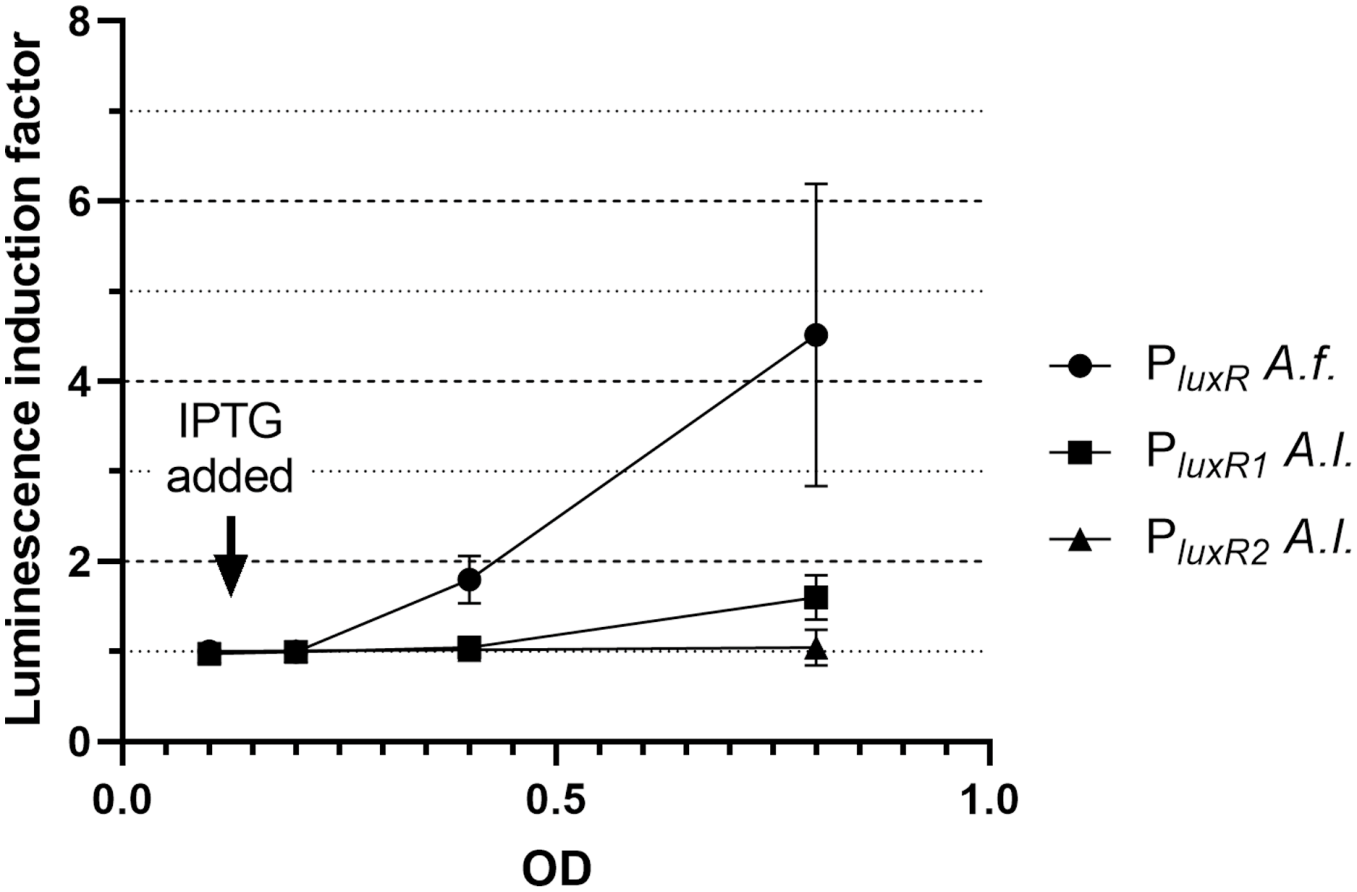
The induction of promoters P*_luxR_ A. fischeri*, P*_luxR1_ A. logei* and P*_luxR2_ A. logei* by enhancement of the *litR A. logei* expression in *E. coli* cells. The curves represent the increase in luminescence of *E. coli* MG1655 cells carrying the *luxCDABE* genes of *P. luminescens* under control of promoter of *luxR A. fischeri* (pAFR plasmid, P*_luxR_* *A.f*. curve), promoter of *luxR1 A. logei* (pALR1 plasmid, P*_luxR1_ A.l*. curve), or promoter of *luxR2 A. logei* (pDewP2rev plasmid, P*_luxR2_ A.l*. curve) in combination with the *litR A. logei* gene under control of P*_lac_* (p15Tc-litR) in response to the addition of one mM IPTG. IPTG was added at OD~0.1.

Firstly, the effect of LitR on the expression of *luxR A. fischeri*, *luxR1 A. logei* and *luxR2 A. logei* was investigated. Figure 2 shows the coefficient of increase in luminescence of biosensors after the activation of the *litR* gene expression.

The data presented in Fig. 2 shows that LitR from *A. logei* is capable of a significant activation of the *luxR A. fischeri* gene transcription. This result is consistent with previous work (Fidopiastis et al., 2002) and means that the LitR homologues from *A. fischeri* and *A. logei* are interchangeable and have a common function. The amino acid sequence of LitR is highly conservative among the *Aliivibrio* species. In particular, LitR proteins from psychrophilic bacteria have almost the same sequences, comparing of them with mesophilic LitR *A. fischeri* gives that DNA-binding regions three compared proteins are identical and whole “helix-turn-helix” domain differs only by several aa substitutions (Fig. S2). Interesting that psychrophilic LitR variants are more ordered than mesophilic one according to analysis with VL-XT, VSL2, VL3 and Charge-Hydropathy instruments at pondr.com (Fig. S3). The increase in LitR content in the cell activates the expression of *luxR1 A. logei*, although to a lesser extent than that of *A. fischeri luxR*, and does not affect the expression of the *luxR2 A. logei* gene. The obtained result was in conflict with the data of Norwegian colleagues on a decrease in the production of an autoinducer in *A. salmonicida litR* mutant cells (Hansen et al., 2015); therefore, it was decided to test the effect of *litR* on the transcription of the “rightward” promoters of *lux* regulon.

### Effect of LitR *A. logei* on promoters of structural genes *luxICDABEG*

**Figure 3:**
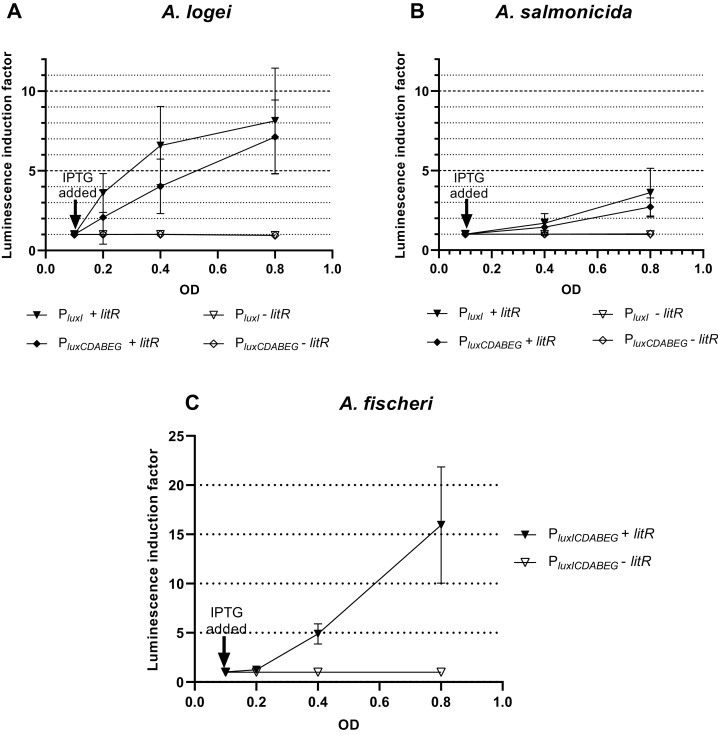
The induction of promoters P*_luxI_* and P*_luxCDABEG_* of *A. logei* (A), *A. salmonicida* (B) and *A. fischeri* (C) by enhancement of the *litR A. logei* expression in *E. coli* cells. Curves represent the increase in luminescence of *E. coli* MG1655 cells carrying the *luxCDABE* genes of *P. luminescens* under control of (A) P*_luxI_ A. logei* (pSV16), P*_luxCDABEG_ A. logei* (pIVA), (B) P*_luxI_ A. salmonicida* (pAS2), P*_luxCDABEG_ A. salmonicida* (pAS1), or (C) P*_luxICDABEG_ A. fischeri* (pVFR1) promoters in combination with the *litR A. logei* gene under control of P*_lac_* (p15Tc-litR) in response to the addition of one mM IPTG at OD~0.1. The “−litR” curve corresponds to a negative control-analogous cell lines, which lacks the *litR* gene (p15Tc-lac vector used).

Results of investigation of LitR *A. logei* effect on expression of autoinducer synthase (*luxI*), luciferase and reductase (*luxCDABE*) genes are given in Fig. 3. Promoters of different *Aliivibrio* species were tested with LitR *A. logei* in the heterological system of *E. coli* cells.

In response to the addition of one mM IPTG at OD~0.1 there was an increase in luminescence of the *E. coli* MG1655 cells carrying combinations of the plasmid p15Tc-litR (*litR* under P_*lac*_) with different biosensor plasmids containing *P. luminescens luxCDABE* genes under control of P_*luxI*_ and P_*luxCDABEG*_ promoters of *A. logei* (Fig. 3A) and *A. salmonicida* (Fig. 3B). The following plasmids with the investigated promoters were used: pSV16 with P_*luxI*_ *A. logei lux*-reporter, pIVA with *A. logei* P_*luxCDABEG*_ *lux*-reporter, pAS2 with *A. salmonicida* P_*luxI*_ *lux*-reporter, and pAS1 with *A. salmonicida* P_*luxCDABEG*_ *lux*-reporter (Table 1, Fig. S1).

As can be seen from the graphs, LitR activates the transcription from the promoters of the *luxI* and *luxCDABEG* genes of both psychrophilic luminescent bacteria species *A. logei* and *A. salmonicida*. It should be noted that in both cases the activation of the *luxI* gene promoter in response to an increased expression of the *litR* gene was somewhat stronger than the activation of the *luxCDABEG* gene promoter. It can be seen that the amplitude of activation of *A. salmonicida* promoters is almost twice lower than that of *A. logei* promoters. This can be explained by the influence of other regulatory elements. These promoters are still the subject of research, but it is already known that their regulation is influenced by many factors, such as Lon, GroEL/ES, H-NS, CRP. We assume that the observed twofold difference may be due to differences in the sequences of promoters and surrounding regions, although the *lux*-box elements for these species are identical.

The data from Fig. 3C indicates that the induction of *litR A. logei* in the heterological system of *E. coli* leads to a notable (more than one order of magnitude) activation of expression from the *A. fischeri* P_*luxICDABEG*_ promoter. This effect has not been previously described and may indicate that *litR* is able to induce LuxI/R QS system through stimulation of both *luxI* and *luxR* gene expressions in *A. fischeri* cells. Moreover, the effect on *luxI* expression was higher in magnitude. The data obtained contradicts the results published by (Fidopiastis et al., 2002), which investigated the effect of *litR* on the expression of *lacZ* of the gene inserted into *luxC* ORF. Perhaps it could be connected with the differences in the sensitivity of the reporter genes used. However, LitR proteins from mesophilic and psychrophilic bacteria have some differences and it needs more detailed investigation.

### LuxR-independent activation of promoters by LitR depends on the *lux*-box sequence

**Figure 4:**
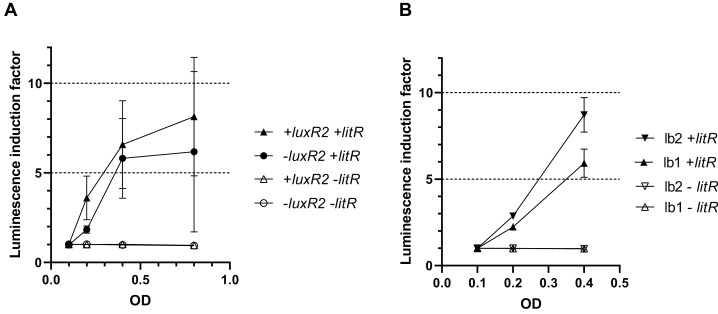
Effect of the *luxR2* gene (A) and the *lux*-box sequence (B) on LitR-mediated activation of the *A. logei lux* regulon promoters in *E. coli* cells. *E. coli* MG1655 cell were used with different combinations of the following plasmids: p15Tc-lac vector (marked as −litR), p15Tc-litR with the *litR* gene under control of P*_lac_* (+litR), pSV16 − P*_luxI_* promoter and *luxR2* gene (+luxR2), pR2 − pSV16 with the truncated *luxR2* gene (marked as –luxR2), pD-lb1 − P*_luxCDABEG_* promoter (lb1), pD-lb2 is pD-lb1 with the *lux*-box sequence from P*_luxI_* (lb2).

To make sure that the observed activation of “rightward” promoters determined by LitR occurs independently of the presence of regulatory genes from the *luxI/R* QS system in the cell, measurements of LitR-dependent activation of *A. logei luxI* gene transcription were carried out in the presence of the *luxR2* regulatory gene and without it. Plasmids pSV16 and pR2 were used (Table 1, Fig. S1), in which the *luxCDABE P. luminescens* genes are under control of the *A. logei* P_*luxI*_ promoter with and without the *luxR2* gene, respectively. The *litR* gene was introduced as before on a separate plasmid p15Tc-litR. Measurement results are shown in Fig. 4A, the increase in luminescence was calculated as the ratio of luminescence of IPTG-induced cells to the luminescence of the same cells without IPTG. As a negative control, cells carrying the p15Tc-lac vector without the *litR* gene were used.

We assumed that LitR could bind DNA in the promoter upstream region, the same site LuxR binds to (*lux*-box). To assess the effect of the *lux*-box sequence on the transcription activation of *A. logei* P_*luxCDABEG*_ by the LitR protein, we compared the LitR-dependent activation of two variants of the *A. logei* P_*luxCDABEG*_ promoter, which differ only in the *lux*-box sequences (Fig. 4B). The native P_*luxCDABEG*_ promoter was truncated exactly upstream of the *lux*-box and introduced into pDEW201 upstream of *P. luminescens luxCDABE* gene cassette (line lb1 on Fig. 4B, the pD-lb1 plasmid was used); to obtain another promoter variant, the *lux*-box was changed to match P_*luxI*_ (lb2 lines on Fig. 4B, the pD-lb2 plasmid was used). The difference between the obtained chimeric promoters is limited to only five bp (Fig. S4), in both cases, the cells don’t contain any of the *luxR*, *luxR1* or *luxR2* genes. All genetic elements, with the exception of *lux*-boxes, were identical in the compared cultures. The *litR* gene, as in previous experiments, was introduced into the cell on the p15Tc-litR plasmid, where it was under control of P_*lac*_.

As can be seen from the data presented in Fig. 4A, LitR activates P_*luxI*_ and P_*luxCDABEG*_ *A. logei* promoters regardless of the presence of LuxR2 in the cells: luminescence induction in response to *litR* expression stimulation is almost equal for cells with *luxR2* gene and without it (p15Tc-litR in combination with pSV16 and pR2, correspondently). The observed induction of P_*luxI*_ and P_*luxCDABEG*_ *A. logei* and consequently luminescence of cells is completely determined by the *litR* gene: in its absence (vector p15Tc-lac in combination with pSV16, pR2, pDlb1, or pD-lb2 biosensor plasmids), the luminescence of cells does not change in response to the addition of IPTG. The data in Fig. 4B shows that the change in the *lux*-box sequence affects the activation of the promoter by LitR. This indicates that the LitR binding site in the promoter region of *A. logei luxCDABEG* genes coincides or at least intersects with the LuxR binding site.

## Discussion

The *luxR-luxI* regulon could be used for development of expression systems with target protein expression regulation in dependence on bacterial population density (Swennen & Nocadello, 2011; Nocadello & Swennen, 2012). *luxI-luxR* regulation is modulated by a large number of intracellular factors that ensure its sensitivity to external conditions (Manukhov, Kotova & Zavil’gel’skiĭ, 2006; Konopleva et al., 2016), LitR becomes on a par with them, giving a connection with other QS systems (Fidopiastis et al., 2002).

Our experiments showed that promoters of *luxI* and *luxCDABEG* genes of all three investigated species of the *Aliivibrio* genus are activated by LitR from *A. logei*. Apparently, the interaction between LuxR and LitR proteins does not occur, since Fig. 4 shows the absence of the effect of the *luxR2* gene on *litR*-mediated activation of the *luxI* gene promoter. That is, the interaction between *litR* and the *lux *operon is realized by the direct influence of LitR on promoters. In the psychrophilic species *A. logei* and *A. salmonicida*, the induction of promoters from which the *luxI* gene is transcribed is notably higher than induction of the other promoters of their *lux* regulons. As LuxI is an autoinducer synthase, its expression induction could stimulate whole QS system. The expression of *luxCDABEG* genes increases in the presence of LitR too, but the amplitude of this induction is too low to make *Aliivibrio* cell luminescence visible. Thus, we assume that the main effect of LitR on *lux* regulons of psychrophilic bacteria of the *Aliivibrio* genus is the independent of LuxR stimulation of LuxI synthesis (Figs. 3 and 4A). When *ainS/R* and *luxS/PQ* QS systems triggered before the *luxI/R* one due to stress conditions, stimulation of LuxI synthesis by LitR brings forth the increase in AI production and gives an initial impulse to activate *luxI/R* system. Higher concentrations of AI stabilize LuxR2 from *A. logei/A. salmonicida*, and the *luxI/R* QS system is triggered (Khrulnova et al., 2016). This LitR-dependent *luxI* induction could appear necessary in stress conditions, when the chaperonin GroEL/ES lacks or the protease Lon content is increased and consequently the QS is not activated even at a high culture density due to the lack of functional LuxR2 (Dolan & Greenberg, 1992a; Zavil’gel’skiĭ & Manukhov, 1994; Khrulnova et al., 2010, 2016).

The LitR binding site is not defined well and is still an object of discussion (Van Kessel et al., 2013; Chaparian et al., 2016). Here we showed that the sequence of *lux*-boxes has significance for the transcription activation of *luxICDABEG* by LitR regardless of the presence of *luxR1* or *luxR2* genes in the cell (Fig. 4). It is an obvious sign that LitR binds to the *lux*-box or directly next to it.

## Conclusion

**Figure 5:**
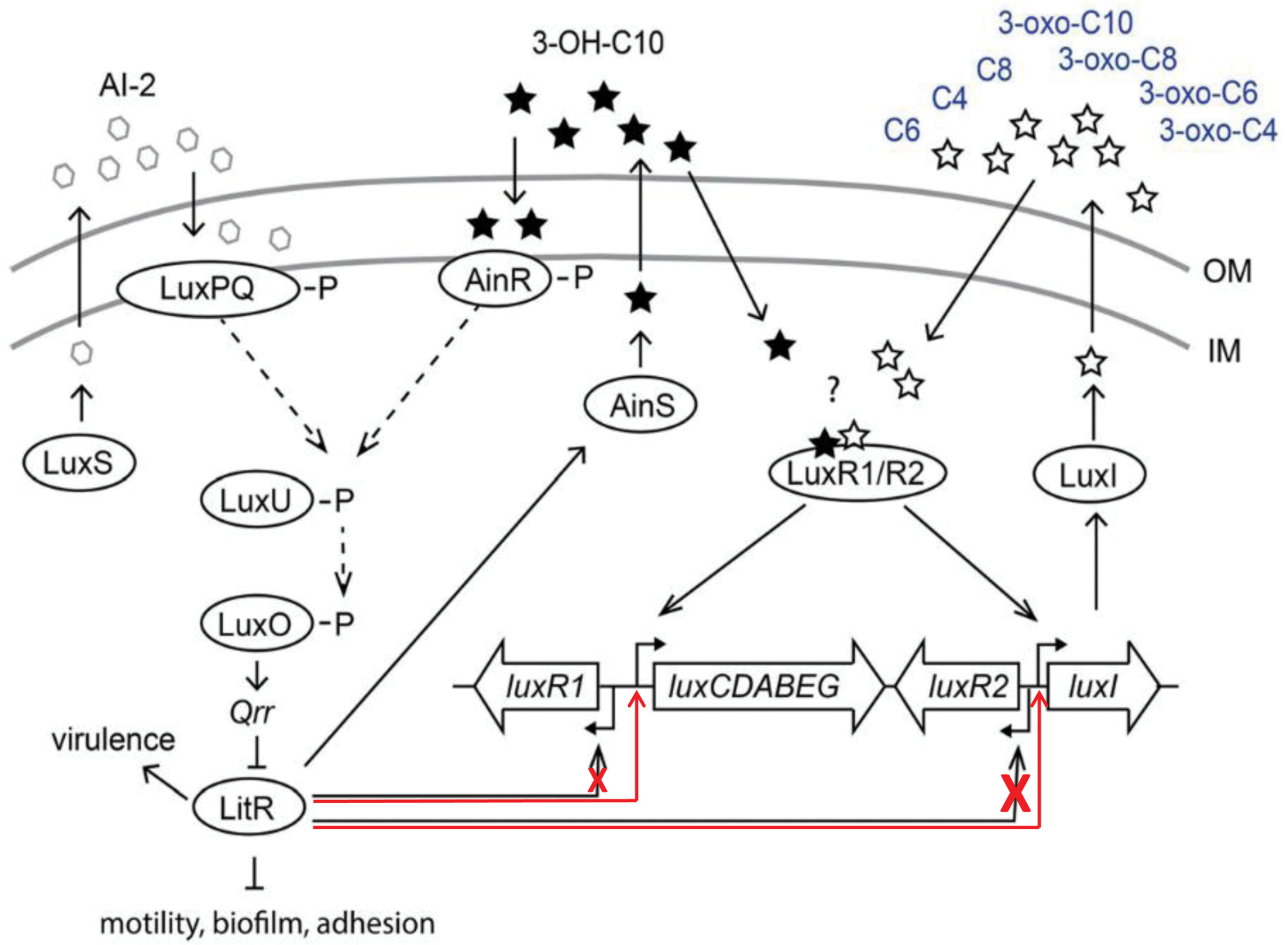
Scheme of *A. logei* and *A. salmonicida* QS regulation. The autoinducer synthases LuxS, LuxI and AinS, produce the different acyl homoserine lactones and autoinducer-2 (AI-2) which are transported across the inner membrane (IM) and the outer membrane (OM). In presence of AI-2 or 3-hydroxo-decanoyl-homoserine lactone (3-OH-C10) LitR synthesis is activated through the AinS/R and LuxS/PQ pathways. LitR is expressed and regulates the production of the AinS AHL, as well as activities such as motility, biofilm, adhesion, virulence and bioluminescence, in accordance with (Bjelland et al., 2012). *A. logei* has the same gene combination in the QS systems as *A. salmonicida*. Red arrows indicate that LitR, contrary to previous assumption, is able to activate *luxI* and *luxCDABEG* genes, but not the *luxR2* (very low effect on the *luxR1* gene). Redrawn from (Hansen et al., 2015).

The results of this study demonstrated that LitR of psychrophilic bacteria *A. logei* is able to directly stimulate (independently of LuxR presence) transcription from promoters of *luxI* and *luxCDABEG* genes and to a lesser degree from promoter of *luxR1* gene. Thus, 3-OH-C10 and AI-2 in the medium induce expression of *litR* and could enhance *luxI* expression and 3-O-C6 synthesis (Fig. 5). But for the effect of 3-OH-C10 and AI-2 on *luxI/R* system to be significant, the conditions are needed preventing this system from normal function. Our finding could be a clue to understanding the cross-interaction of the *luxS/PQ* and *ainS/R* QS systems with the *luxI/R* one in psychrophilic bacteria of *A. logei* and *A. salmonicida* species. Such effect was not previously described for the *luxI/R* QS system of well-known mesophilic bacteria *A. fischeri*. Main effect of this cross-interaction is LitR-dependent stimulation of autoinducer synthesis through P_*luxI*_ induction and this pathway could be activated in conditions, when *luxI/R* QS system was not activated by its own AI until high culture density.

## Supplemental Information

10.7717/peerj.12030/supp-1 Supplemental Information 1 Supplementary materials, Supplemental Figures, and plasmids constructing description.

10.7717/peerj.12030/supp-2 Supplemental Information 2 Raw data of measurements. Measurements of luminescence of *E. coli* cells transformed promoter-reporter plasmids in combination with plasmid carrying *litR A. logei* gene under control of Plac promoter. Luminescence was used for calculation of induction coeffitients.

10.7717/peerj.12030/supp-3 Supplemental Information 3 p15Tc-litR plasmid sequence.

## Additional Information and Declarations

### Data Availability

The following information was supplied regarding data availability: The raw measurements of luminescence are available in the Supplementary File.
