# Supplementary material for: LitR directly upregulates autoinducer synthesis and luminescence in Aliivibrio logei
Source: PeerJ. 2021 Sep 21;9:e12030. doi: 10.7717/peerj.12030 (PMC8462370; doi:10.7717/peerj.12030)
Supplement: Supplemental Information 1 [file peerj-09-12030-s001.docx]

**Supplementary Materials for:**

**LitR upregulates *luxICDABE* genes of bacteria from *Aliivibrio* genus.**

**Bazhenov S.V., Melkina O.E., Fomin V.V., Scheglova E.S., Krasnik P.V., Khrulnova S.A., Zavilgelsky G.B., Manukhov I.V.**

**
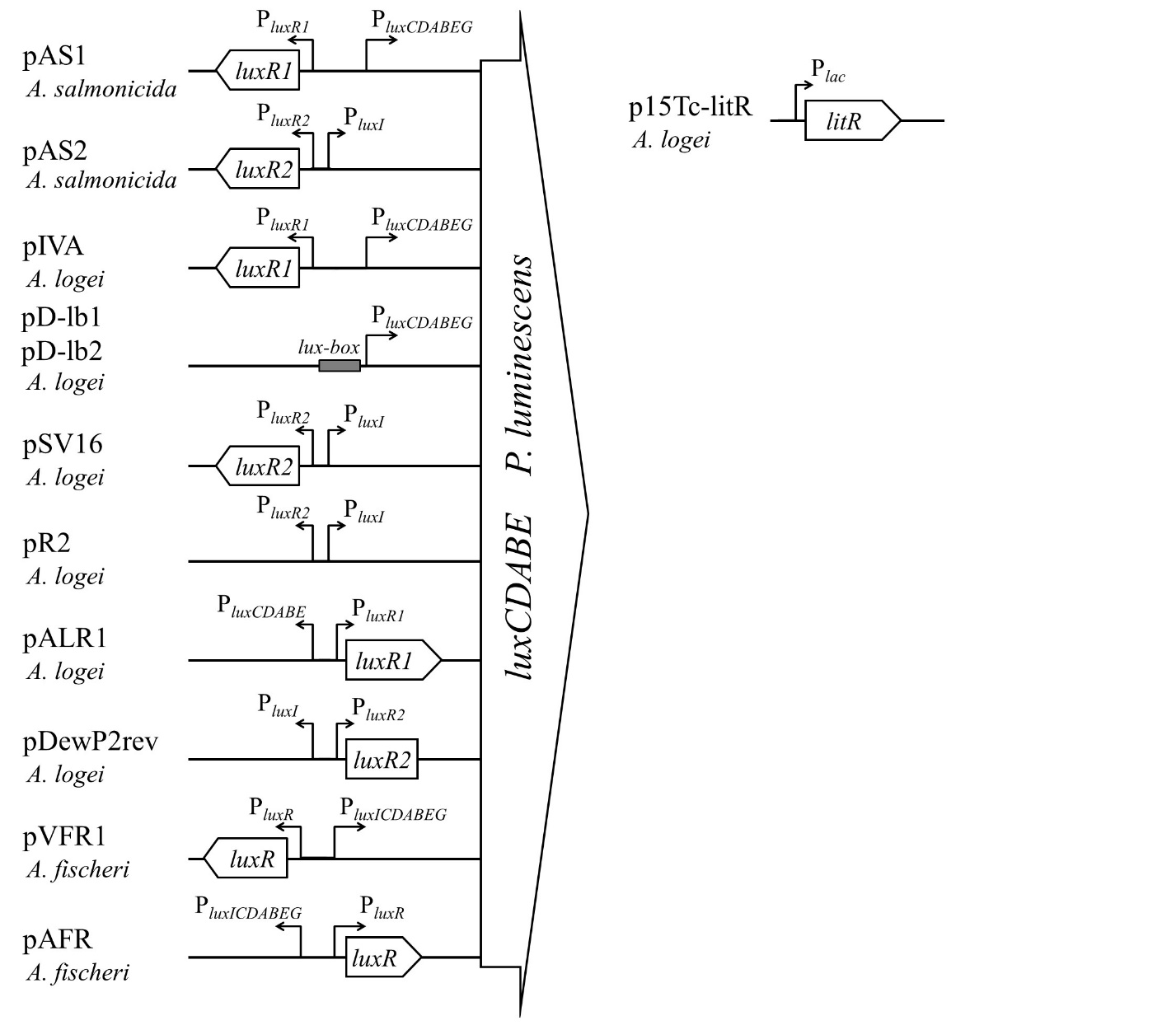
**

**Figure S1** Schemes of used in the study plasmids. Left part of the figure shows promoters and genes being inserted in the pDEW201 promoter probe vector with *luxCDABE Photorhabdus luminescens* genes with references to biosensor plasmids names. pD-lb1 and pD-lb2 differ only by 5 bp in *lux*-box. Right part of the figure shows p15Tc-litR.


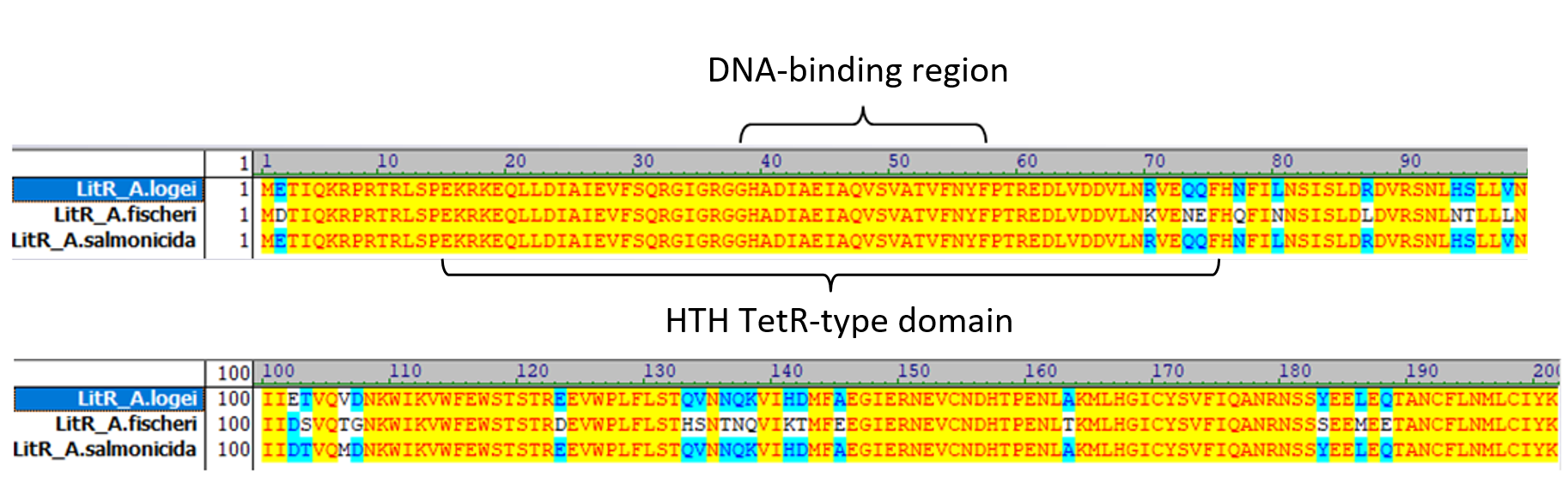


**Figure S2** Alignment of amino acid sequences of LitR of *Aliivibrio logei, Aliivibrio fischeri*, and *Aliivibrio salmonicida*. DNA-binding region (38-57) and HTH domain are marked with bracket according to UniProt data (A0A1B9P2H3_ALILO, Q5E2S4_ALIF1, and B6ELF1_ALISL, correspondently)


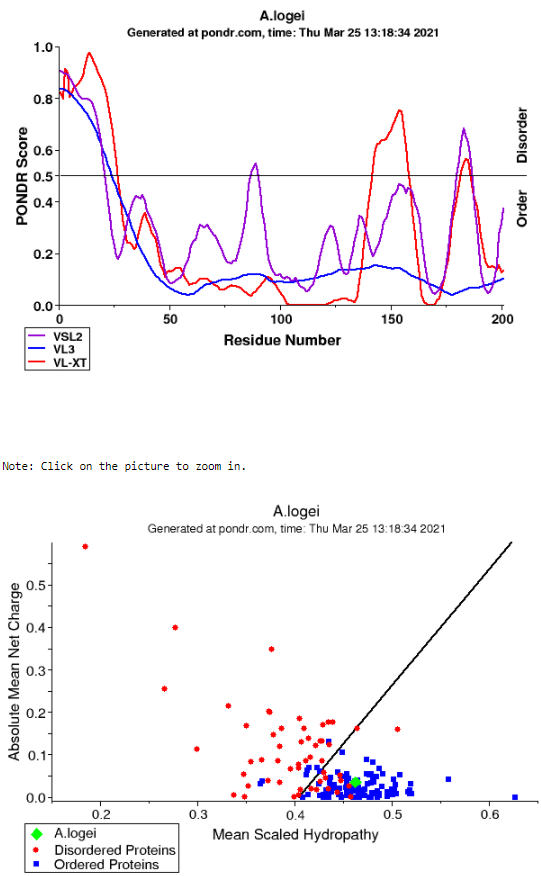

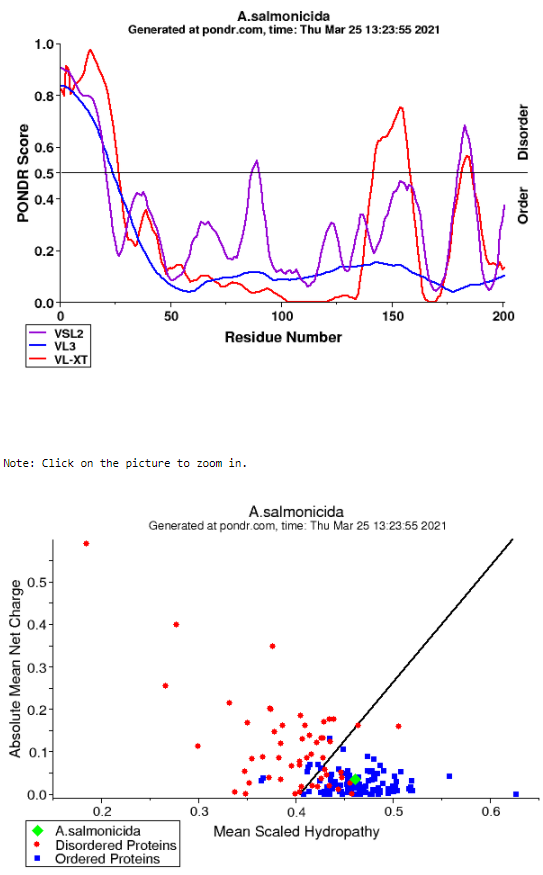

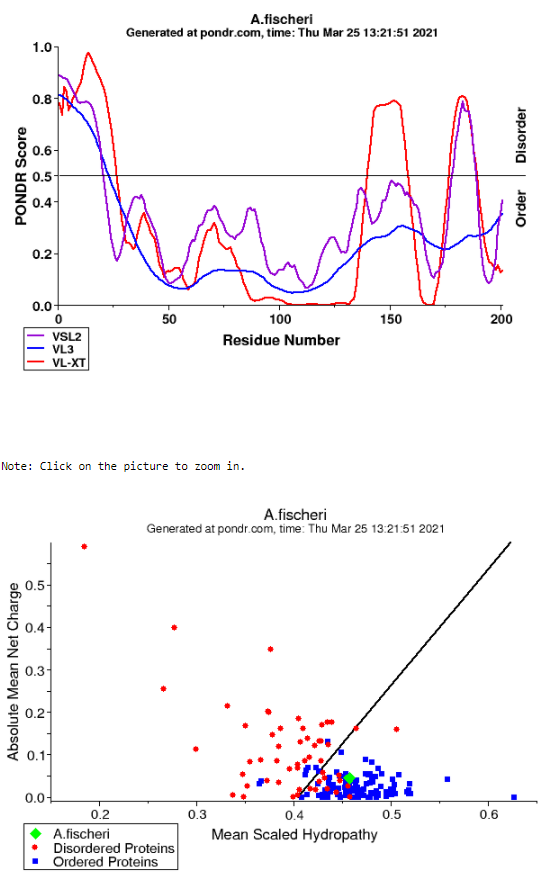


**Figure S3** Analysis of natural disordered regions of LitR proteins from *A. logei, A. fischeri, and A. salmonicida* with VL-XT, VSL2, VL3, and Charge-Hydropathy instruments at pondr.com (A0A1B9P2H3_ALILO, Q5E2S4_ALIF1, and B6ELF1_ALISL, correspondently)


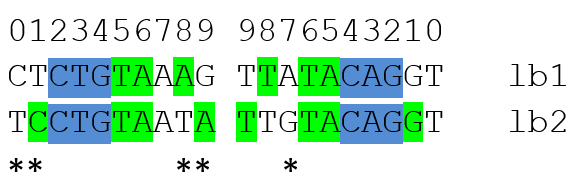


**Figure S4** Alignment of nucleotide sequence of *lux*-boxes from *A. logei* P*_luxCDABEG_* (lb1) and P*_luxI_* (lb2). *lux*-box is 20-bp inverted repeat in promoter upstream region *- Aliivibrio* LuxR binding site. Dark blue highlights core positions essential for LuxR binding, green – palindromic positions. Stars marks 5 positions, in which *lux*-boxes from *A. logei* P*_luxCDABEG_* and P*_luxI_* differ.

**Plasmid constructing**

p15Tc-litR plasmid (sequence in GenBank format is given as separate file in Supplementary) was constructed on base of p15Tc-lac vector (Bazhenov et al., 2021), *litR* gene was amplified with primers litRDEco and litRRKpn from gDNA of *A. logei* Skal and inserted in EcoRI/KpnI restriction sites of p15Tc-lac.

pAS1 and pAS2 plasmids were obtained by introducing into pDEW201 promoter-probe vector by EcoRI/BamHI restriction sites of two fragments of *A. logei* KCh1 gDNA: (1) *luxR1* gene with P*_luxR1_* and P*_luxCDABEG_* promoters and (2) *luxR2* gene with P*_luxR2_* and P*_luxI_* promotes. Fragment (1) was amplified with use of luxR1(end) and SV1dir primers, fragment (2) ― luxR2(RevSt) and revpl1 primers. The PCR products were cloned into pTZ57R/T vector (Fermentas, USA) and then were transferred to pDEW201 by EcoRI and BamHI restriction sites.

To obtain the pR2 and pDewP2rev plasmids DNA fragment of KCh1 gDNA, containing divergent P*_luxI_* and P*_luxR2_* promoters, was amplified with use of P2Irev and P2R2rev primers and then was introduced into EcoRI site of pDEW201. Two opposite orientations of insertion gave two lux-reporter plasmids: lux-reporter for *A. logei* P*_luxI_* where *P. luminescens luxCDABE* genes are fused with P*_luxI_*; and lux-reporter for P*_luxR2_*, correspondently.

For constructing of pDlb1 plasmid P*_luxCDABEG_* promoter of *A. logei* KCh1 was amplified with use of luxCrevR1 and lb1Dir primers, subcloned into pTZ57R/T vector and transferred to pDEW201 into EcoRI/BamHI restriction sites upstream from *P. luminescens luxCDABE*. To obtain pDlb2 plasmid the same operations were done, but luxCrevR1 and lb2Dir primers were used. Use of lb1Dir or lb2Dir primers generate two variants of the lux-box sequence (Figure S4) in cloned P*_luxCDABEG_* promoter in pDlb1 and pDlb2.

**Table S1** Oligonucleotides used for cloning promoters into promoter-probe vector and litR under P*_tac_* promoter in this study.

Nucleotides, which are complementary to matrix, are given in bold, 5’ region of most primers have adapters for cloning through Gibson Assembly or restriction/ligation.

| Primer | Sequence, 5’-3’ |
| --- | --- |
| litRDEco | GAATTC**ATGGAAACGATCCAAAAAAGACCGA** |
| litRRKpn | GGTACCA**TTATTTATAAATACACAGCATATTTAAGAAGCAG** |
| lb1Dir | **GATACTCTGTAAAGTTATACAGGTTTACCTA** |
| lb2Dir | **GAT**TTC**CTGTAA**TA**TT**G**TACAGGTTTACCTAAATAATTACCCTGCTA** |
| luxCrevR1 | **GACACCGCCGATGATAATTGGA** |
| P2Irev | GAATTC**CCTGACCCCCTTTAATCTTTTAACTG** |
| P2R2rev | GAATTC**GTAGTTATCTAAAATTTTAATATCAGATTTGATCATGG** |
| luxR2(RevSt) | **TGAAAATTAGAAGTCCGACTGCGT** |
| Revpl1 | **GTCATCCTGACCCCCTTTAATCTTT** |
| SV1dir | **TCACACCGCCGATGATAATTGGAA** |
| luxR1(end) | **GGCCGAATTGTATGTAAAAATAAATGAG** |
